# Real-world pharmacovigilance analysis of drug-related cataracts using the FDA adverse event reporting system database

**DOI:** 10.3389/fphar.2025.1498191

**Published:** 2025-04-24

**Authors:** Xiang Li, Shu Wen Wang, Zhi-Jie Zhang, Zhan Yang Luo, Jia Feng Tang, Tao Tao

**Affiliations:** ^1^ Eye Institute & Affiliated Xiamen Eye Center, School of Medicine, Xiamen University, Xiamen, Fujian, China; ^2^ Chongqing Key Laboratory of Development and Utilization of Genuine Medicinal Materials in Three Gorges Reservoir Area, Chongqing Three Gorges Medical College, Wan Zhou, China; ^3^ Shanghai Pudong Hospital, Fudan University Pudong Medical Center, Shanghai, China

**Keywords:** FAERS, cataracts, drug, cataract prevention, adverse event

## Abstract

**Objective:**

Although numerous drugs have been associated with cataracts, the risk for most drugs remains unclear. This study aimed to investigate the risk factors for drug-induced cataracts by analyzing large-scale data from the U.S. Food and Drug Administration Adverse Event Reporting System (FAERS).

**Methods:**

We used the reporting odds ratio (ROR) to evaluate reports of drug-induced cataracts in FAERS from the first quarter of 2004 to the third quarter of 2024. A univariate analysis, LASSO (least absolute shrinkage and selection operator) regression, and a multivariate regression analysis were performed to identify drug-related risk factors for cataracts, and Bonferroni correction was applied for multiple comparisons.

**Results:**

Multivariate logistic regression ultimately identified 15 drugs as independent risk factors, including immunomodulators (6/15), antineoplastic drugs (3/15), psychotropic drugs (1/15), respiratory drugs (1/15), gastrointestinal drugs (1/15), orthopedic drugs (1/15), metabolic regulators (1/15), and ophthalmic drugs (1/15). The median time to onset of drug-induced cataracts was 449 days (interquartile range [IQR]: 150–901 days), with approximately 75% of adverse events occurring within 747 days.

**Conclusion:**

These findings may help clinicians detect drug-related cataracts at an early stage and provide valuable insights for future research on the mechanisms of drug-induced cataracts.

## Graphical Abstract

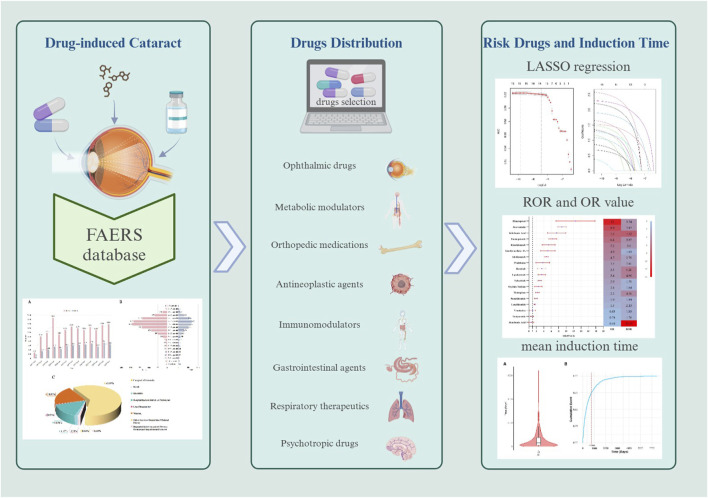

## Introduction

The eye lens is an optically transparent structure situated posterior to the iris and anterior to the vitreous body and retina. Its distinctive morphology and refractive properties enable the precise focusing of light onto the retina (Thakur and Panda, 2000). With advancing age, the eye lens gradually becomes denser and thicker. Cataracts develop when eye lens transparency is compromised, typically manifesting clinically as decreased visual acuity, reduced contrast sensitivity, impaired color perception, and heightened glare sensitivity (Tewari et al., 2019). Common risk factors for cataract formation include aging, smoking, ultraviolet radiation exposure, diabetes mellitus, ocular trauma, and adverse drug reactions (Congdon, 2001). Additionally, certain medications may induce drug-related cataracts through mechanisms primarily involving direct drug toxicity, metabolic disturbances within the eye lens, and oxidative stress reactions (Szostakiewicz-Grabek et al., 2016). While most drug-induced cataracts result from prolonged systemic medication use (e.g., glucocorticoids), rare cases have been reported following chronic topical ocular application of glucocorticoid-containing eye drops.

Although cataracts are generally considered treatable, they remain one of the leading causes of blindness worldwide (Hashemi et al., 2009). Cataracts continue to pose a significant public health challenge in ophthalmology, affecting both developed and developing countries. The World Health Organization (WHO) estimates that approximately 180 million individuals worldwide experience some degree of visual impairment, with cataracts accounting for approximately 46% of blindness cases (GBD, 2019 Blindness and Vision Impairment CollaboratorsVision Loss Expert Group of the Global Burden of Disease Study, 2021). The burden of cataract-related diseases is expected to escalate further due to global population growth and aging.

Although drug-induced cataracts are clinically prevalent, systematic research on this condition remains insufficient, with existing knowledge primarily derived from case reports. This research gap limits a comprehensive understanding of cataract pathogenesis, epidemiological characteristics, and clinical management strategies. Furthermore, as novel medications continue to emerge and drug administration routes diversify, the pharmacological spectrum and clinical manifestations of drug-induced cataracts may evolve accordingly. This underscores the need for continuous pharmacovigilance and in-depth research to refine and update clinical evidence and practice guidelines.

Currently, a clinical consensus exists regarding the cataractogenic risks associated with both local and systemic drug use, particularly with glucocorticoids (Szostakiewicz-Grabek et al., 2016), anti-neoplastic agents (Uboweja et al., 2006), psychotropic medications (Fang et al., 2019), and statins (Mach et al., 2018). However, systematic research on drug-induced cataracts remains limited. With the ongoing introduction of new drugs and the diversification of administration routes, the spectrum of cataractogenic medications and their clinical presentations is expected to change. This highlights an urgent need for sustained pharmacovigilance and in-depth investigations to enhance the existing knowledge base and improve patient safety.

Given the limitations of previous research, the analysis of adverse drug events using large-scale databases holds significant practical implications for clinical practice. FAERS serves as a comprehensive and information-rich pharmacovigilance database, collecting spontaneous post-marketing reports of adverse drug events associated with medical products (Li et al., 2025a). As a crucial tool for drug safety monitoring, FAERS provides essential data for epidemiological studies of adverse drug reactions and post-marketing surveillance. In this study, we systematically evaluate the occurrence of drug-induced cataracts using extensive real-world data, encompassing both topical ocular and systemic drug applications. Additionally, this study may reveal previously unidentified associations between specific medications and cataracts, offering novel insights for improving drug safety management.

## Methodology

### Research design and data sources

This study is a retrospective observational pharmacovigilance analysis based on the FAERS. The research methods and procedures strictly follow the relevant guidelines and standards set forth in Recommendations for the Evaluation of Adverse Drug Reaction Signals Using Individual Case Safety Reports (READUS-PV) (Fusaroli et al., 2024). Because the FAERS database is publicly available and all patient information is anonymized, this study does not require ethical approval or patient informed consent.

### Data extraction and processing

**FIGURE 1:**
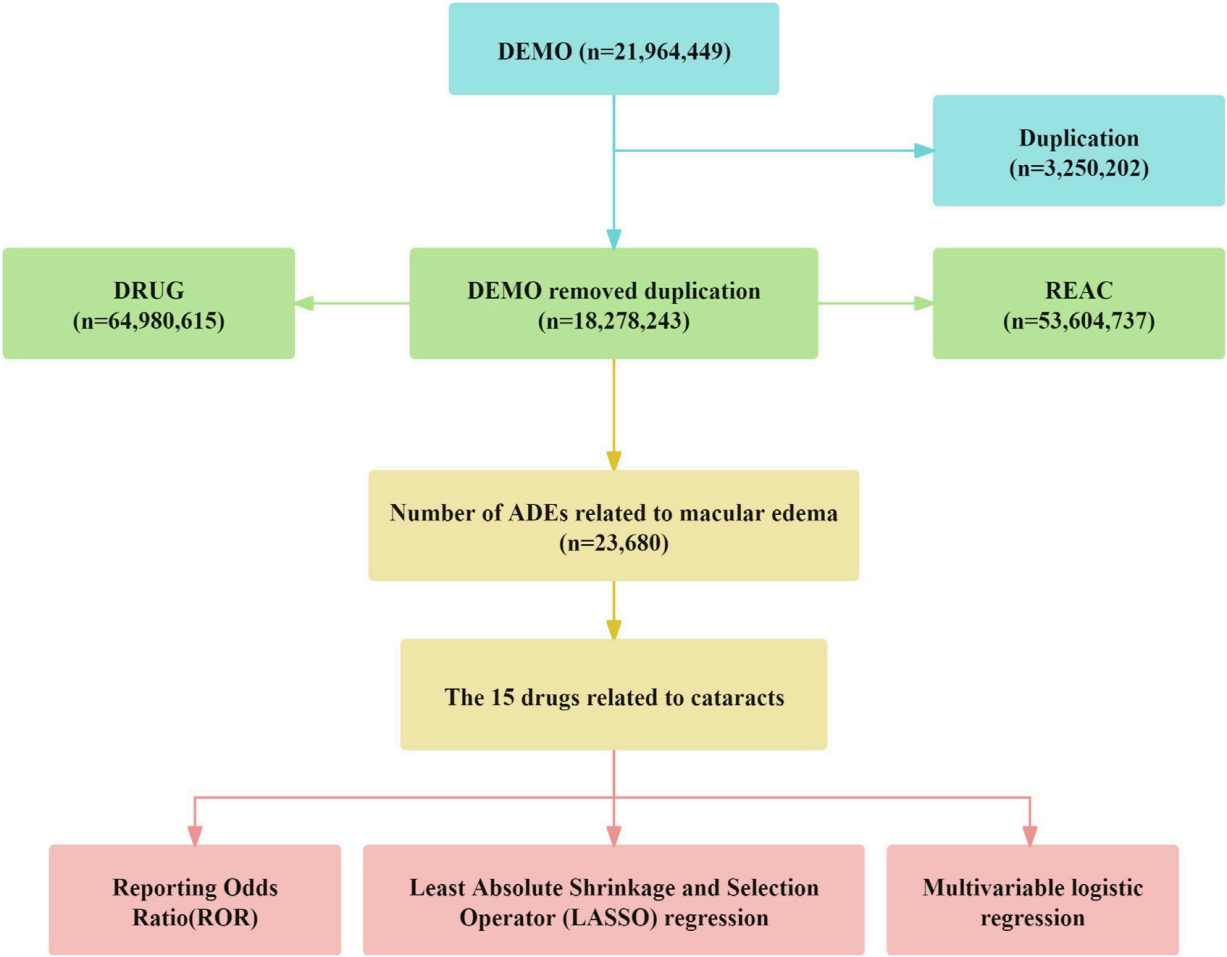
The flow diogram of screening reports containing Cataracts elicited by diverse agents from the FAERS database.

All adverse events were systematically coded using the internationally recognized and clinically validated Medical Dictionary for Regulatory Activities (MedDRA). Employing Open Vigil 2.1–MedDRA software, we searched for adverse event reports related to “cataract” and extracted detailed information based on Primary Identifier (PRIMARYID), including individual case safety reports (ISRs), demographic data (patient age, sex, reporting country, etc.), adverse event descriptions, medication usage, reporting date, and clinical outcomes. Between January 2004 and September 2024, FAERS recorded a total of 21, 964, 449 initial reports. After deduplication, 18, 278, 243 records were included. Through further consolidation of duplicate brand-name drugs, 1,117 distinct drugs were identified. The detailed process of data screening and cleaning is illustrated in Figure 1. To reduce uncertainty and ensure specificity, this study included only drugs classified as “primary suspected.” Cases concurrently labeled as concomitant medications, secondary suspected drugs, or interacting drugs were excluded. Regarding demographic information, only cases with complete age, sex, and weight data were retained, while cases in which the reported age exceeded 120 years or weight was over 400 kg were excluded.

### Statistical analysis

Individuals who used the target drugs constituted the experimental group, while those who used non-target drugs served as the control group, forming a 2 × 2 contingency table for analysis (Supplementary Table 1). We used the ROR and its corresponding 95% confidence interval (CI) to conduct disproportionality analysis, aiming to evaluate the association between the drugs and cataracts while excluding medications specifically intended for cataract treatment. A potential risk signal was considered present if the ROR exceeded 3 and the lower limit of the 95% CI was greater than 1 (Li et al., 2025b). The calculation formula is as follows:

$$ROR=\frac{a/c}{b/d}{ROR}_{95\%CI}=e^{\ln\left(ROR\right)\pm 1.96\sqrt{\left(\frac{1}{a}+\frac{1}{b}+\frac{1}{c}+\frac{1}{d}\right)}}$$

In this study, we extracted complete data on patient sex, age, and weight from FAERS, including only cases with all required data. Patients who were older than 120 years or weighed more than 400 kg were excluded. First, we performed a univariate analysis using the ROR. Drugs were selected if the 95% CI lower limit was greater than 1, the ROR exceeded 3, and the adjusted p-value was below 0.01.

Subsequently, drugs with p < 0.01 in the univariate analysis were included in a LASSO regression model for variable selection. We then conducted a multivariable logistic regression analysis, incorporating demographic variables, to further assess the independent association between specific drugs and cataracts.

Finally, demographic characteristics (sex, age, and weight) were analyzed after excluding extreme values (age >120 years or weight >400 kg). All statistical analyses were carried out using R software (version 4.2.2), and data extraction and processing were performed with Open Vigil 2.1–MedDRA. The univariate analysis included drugs with ROR >3, a 95% CI lower limit greater than 1, and a significance threshold of p < 0.01. All statistical analyses were conducted using R software (version 4.2.2) and Post-Ingres Structured Query Language (PostgreSQL).

## Results

### Baseline characteristics of cataract patients

**FIGURE 2:**
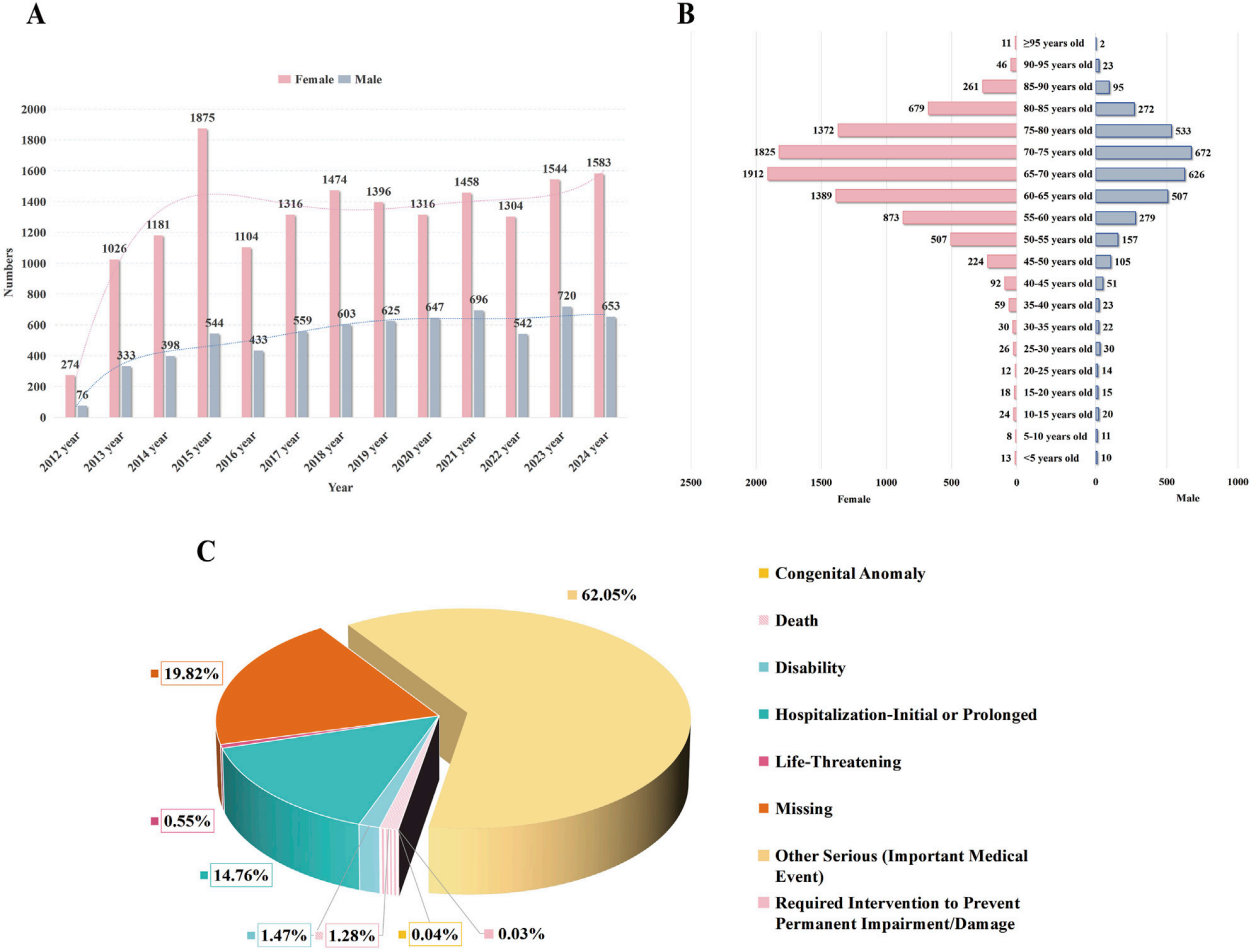
Characteristics of reports involved in drug-indued cataracts from the FAERS database. Notes: **(A)** displays a timeline chart showing the distribution of reported adverse events of cataracts over time. **(B)** depicts a pyramid chart of age distribution among patients reporting adverse events of cataracts, categorized by gender. **(C)** illustrates a pie chart representing the distribution of outcomes among patients experiencing adverse events of cataracts.

A total of 23,680 patients with cataract-related adverse event reports were included in this study. The number of reports peaked in 2015 (n = 2,419), exhibiting an overall increasing trend over the years (Figure 2A). Notably, a significant gender disparity was observed, with female patients accounting for 71.2% of the cases (Figure 2B). The mean age of patients with drug-related cataracts was 66.92 ± 11.95 years. The age distribution trends were similar between genders, with most reports concentrated in the 65–70 age group (females: 1,912 cases; males: 626 cases). Regarding clinical outcomes, 62.05% of patients (n = 14,693) experienced serious medical events (Figure 2C). The detailed information is shown in Table 1.

**TABLE 1:** Baseline data of cataracts patients reported in the FAERS database.

	Variables	Value
	Age (year)	66.92 ± 11.95
	Weight (kg)	72.44 ± 22.53
Gender		
	Female	16,851 (71.16%)
	Male	6,829 (28.84%)
Outcome		
	Congenital Anomaly	9 (0.04%)
	Death	302 (1.28%)
	Disability	348 (1.47%)
	Hospitalization-Initial or Prolonged	3,496 (14.76%)
	Life-Threatening	130 (0.55%)
	Missing	4,693 (19.82%)
	Other Serious (Important Medical Event)	14,694 (62.05%)
	Required Intervention to Prevent Permanent Impairment/Damage	8 (0.03%)
Country		
	United States	23,680 (100%)

Notes: Continuous numerical variables are expressed as mean ± standard deviation, and categorical variables are presented as n (%).

### Drugs associated with cataracts

**FIGURE 3:**
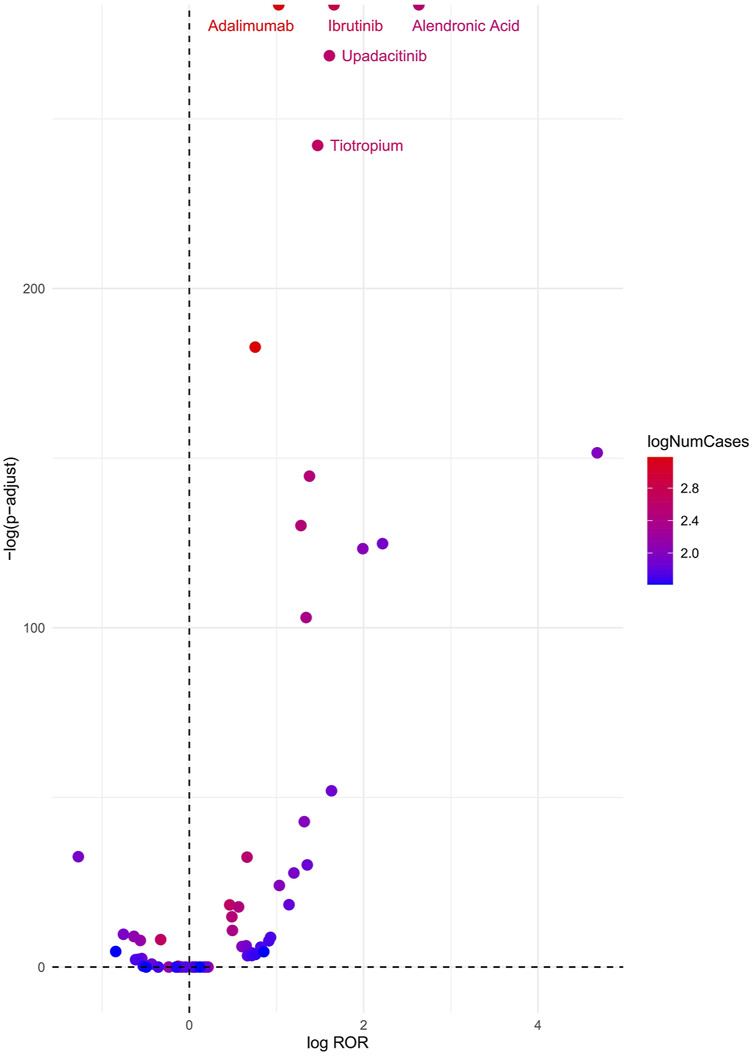
Cataracts-related drug volcano plots. Notes: ROR, reporting odds ratio; P-adjust, p-value after Bonferroni correction.

A volcano plot was constructed to evaluate the potential associations between medications and cataracts (Figure 3). In this plot, the x-axis represents the logarithmic ROR, where higher values indicate a stronger proportional association between adverse event reporting and cataracts. The y-axis represents the negative logarithm of the adjusted P-value (P-adjusted) after Fisher’s exact test with Bonferroni correction, with higher values signifying greater statistical significance. The color of each point corresponds to the logarithmic number of reported cases, with redder shades indicating a higher number of cases. Therefore, medications positioned in the upper-right quadrant of the volcano plot exhibit stronger signal intensity and higher statistical significance.

According to the analysis, the top five medications associated with an increased risk of cataracts were Alendronic Acid (ROR = 13.92, 95% CI: 12.51–15.48, P < 0.01), Ibrutinib (ROR = 5.26, 95% CI: 4.85–5.70, P < 0.01), Upadacitinib (ROR = 4.99, 95% CI: 4.52–5.51, P < 0.01), Tiotropium (ROR = 4.36, 95% CI: 3.97–4.97, P < 0.01), and Adalimumab (ROR = 2.79, 95% CI: 2.65–2.94, P < 0.01).

### Risk factors for drug-related cataracts

**FIGURE 4:**
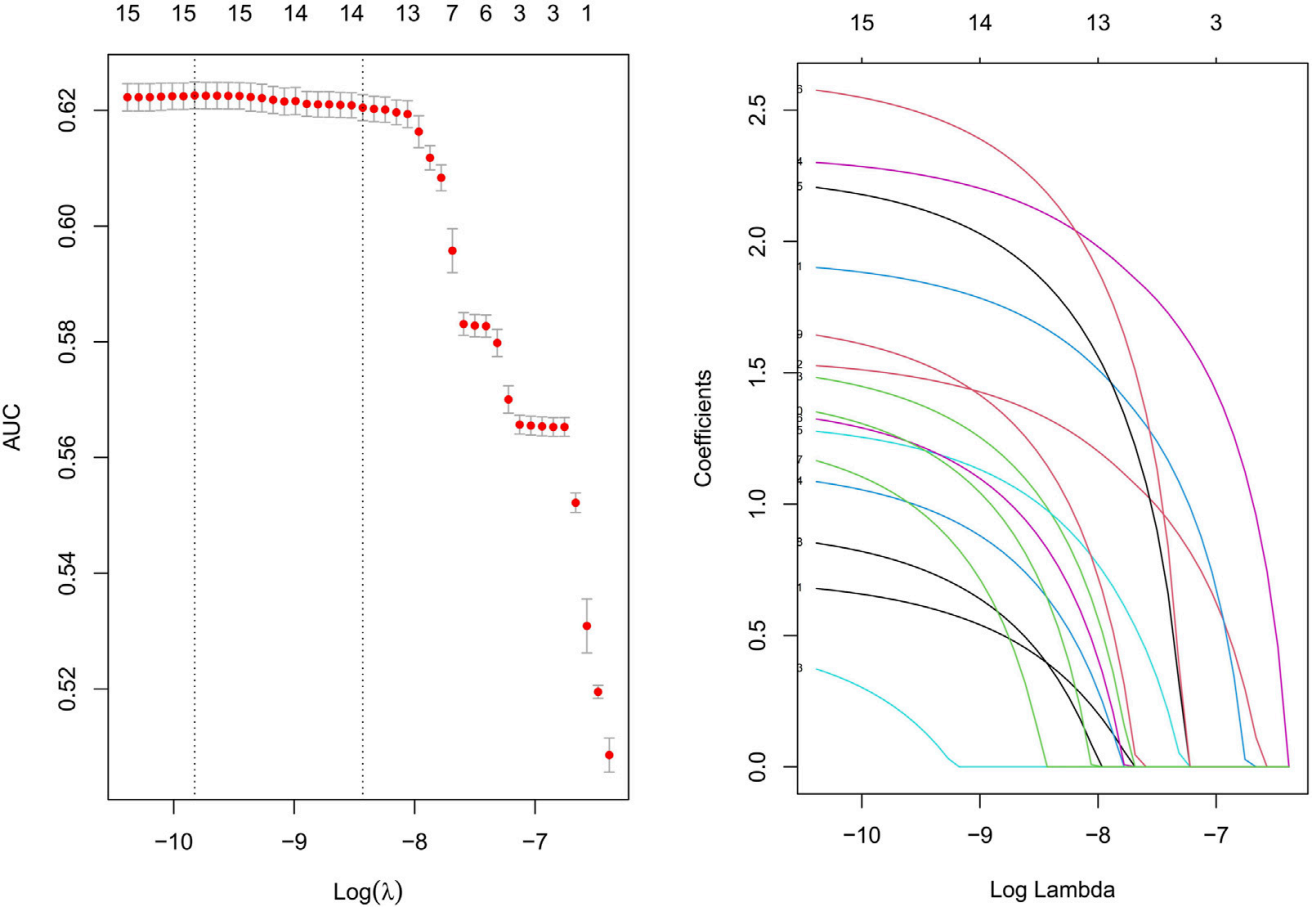
Results of the LASSO regression analysis. Note: LASSO, least absolute shrinkage and selection operator.

This study performed univariate analysis on medications reported in more than 100 cases, selecting those with a lower bound of the 95% CI for ROR greater than 1 and a Bonferroni-adjusted P-value (P-adjusted) < 0.01. Subsequently, medications with P < 0.01 in the univariate analysis were further screened using a Least Absolute Shrinkage and Selection Operator LASSO regression model, identifying 18 candidate drugs (Figure 4).

**FIGURE 5:**
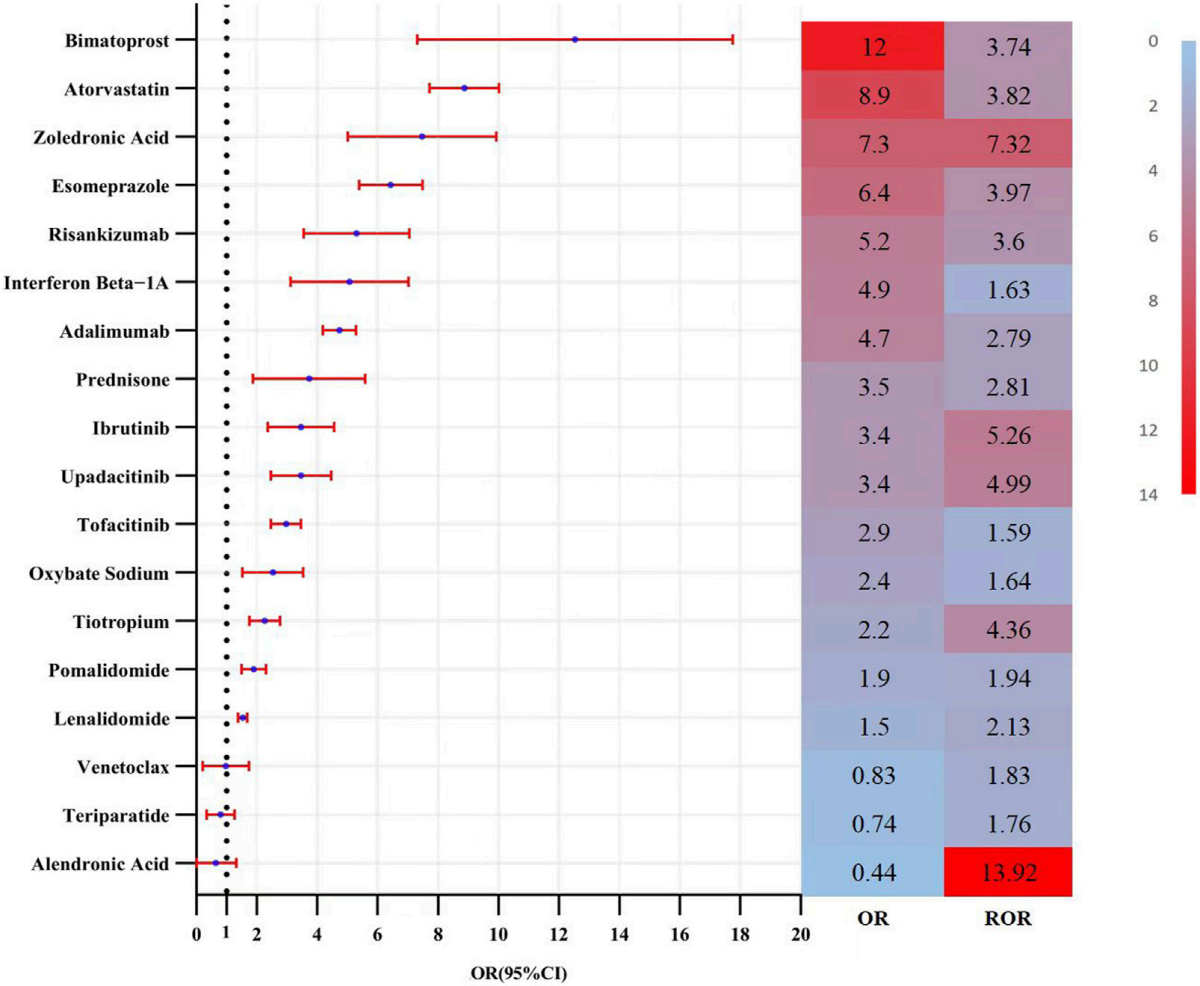
Results of the multi-factor logistic regression analysis and ROR value. Note: CI, confidence interval; OR, odds ratio; P-adjust, p-value after bonferroni correction; P-adjust <0.01, statistically significant. ROR, Reporting Odds Ratio.

**FIGURE 6:**
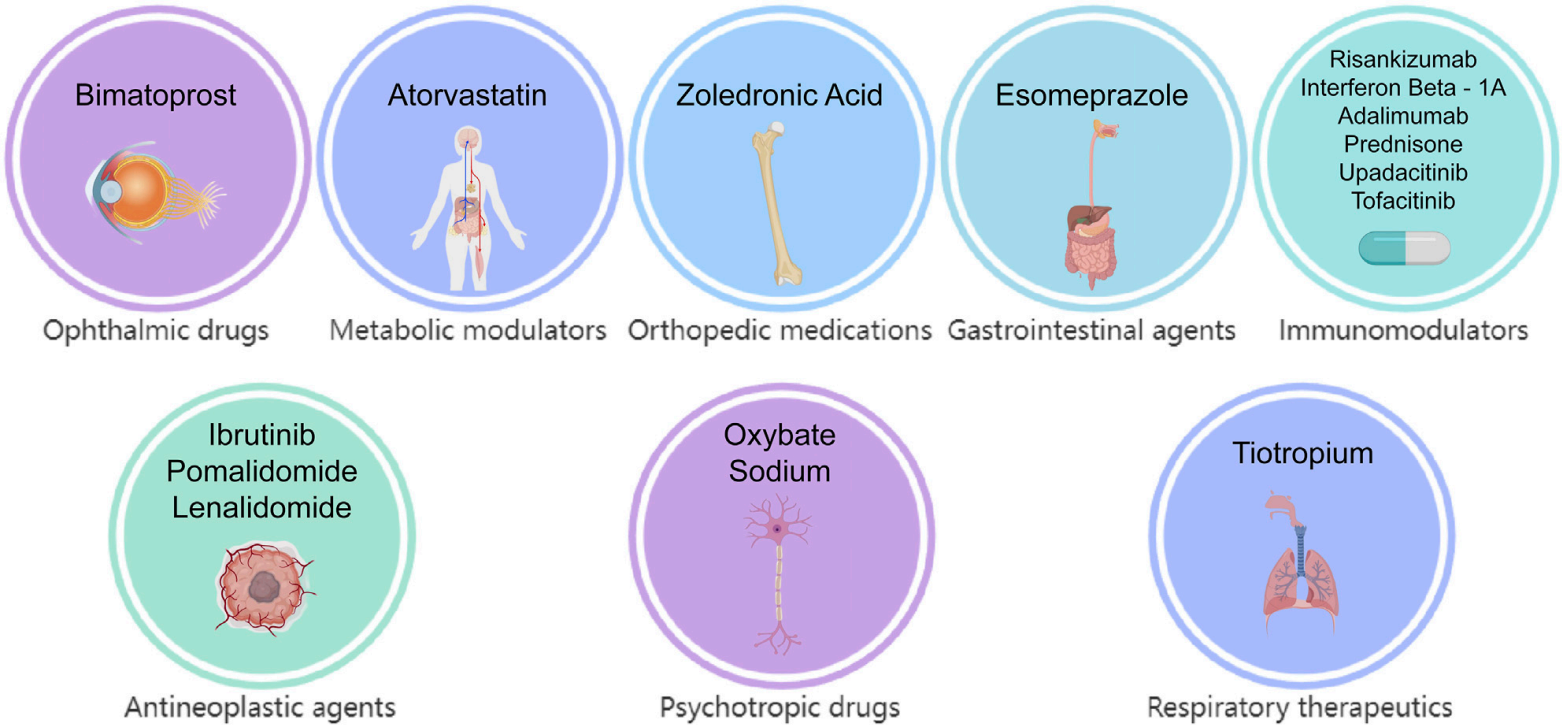
The identification and classification of drugs associated with cataracts.

Multivariate logistic regression analysis, incorporating demographic data, was conducted for these candidate medications (Figure 5). The results identified 15 drugs (Bimatoprost, Atorvastatin, Zoledronic Acid, Esomeprazole, Risankizumab, Interferon β-1, Adalimumab, Prednisone, Ibrutinib, Upadacitinib, Tofacitinib, Sodium Oxybate, Tiotropium, Pomalidomide, and Lenalidomide) as independent risk factors for drug-induced cataracts. Based on pharmacological characteristics, these medications were further classified into eight distinct drug categories (Figure 6).

**FIGURE 7:**
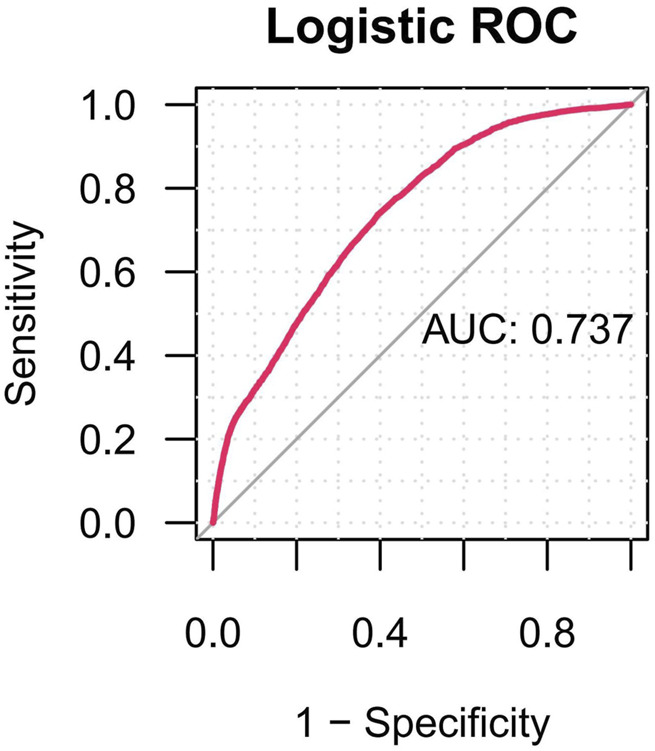
The ROC curves of drug-related cataracts risk factors. Notes: ROC, receiver operating characteristic; AUC, area under curve.

The predictive performance of the multivariate logistic regression model yielded an area under the ROC-AUC of 0.737 (Figure 7).

### Onset time

**FIGURE 8:**
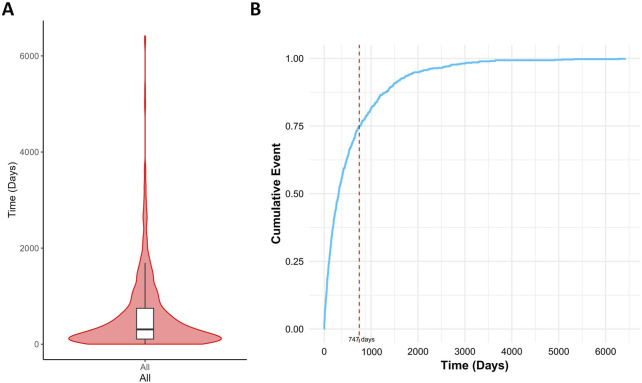
Time interval between drug intake and the onset of drug-related cataracts. Notes: **(A)** Violin plot of time to drug-related cataracts occurrence; **(B)** Cumulative incidence of drug-related cataracts.

This study also analyzed the time interval between medication use and cataract onset. The results indicated a median onset time of 449 days (interquartile range: 150–901 days) for drug-induced cataracts, with approximately 75% of cases occurring within 747 days after medication initiation (Figure 8).

## Discussion

Drug-induced cataracts are increasingly recognized as significant adverse events associated with various medications (Zhao et al., 2021). In recent years, a growing number of case reports have documented drug-induced cataracts, correlating with the expanding use of medications and ongoing drug development. This trend has raised awareness of drug-induced cataracts; however, public awareness of ocular adverse drug reactions remains insufficient.

This study systematically reviewed anonymized cataract case reports from FAERS, comprehensively identifying medications potentially associated with cataract risk. By leveraging this extensive real-world database, disproportionality analysis, univariate analysis, and multivariate regression analysis identified 15 drugs as independent risk factors for drug-induced cataracts, thereby providing critical insights into medication-related cataract risk.

Notably, the proportion of female patients (71.16%) was significantly higher than that of male patients, possibly reflecting longer female life expectancy, estrogen fluctuations during menopause, and greater visual burden in daily life (Zou et al., 2024). Consequently, particular attention should be given to females when assessing cataract risk associated with medication use.

The 15 identified medications were classified into eight drug categories, with ophthalmic drugs being the first group, represented by Bimatoprost. Bimatoprost, a prostaglandin analog commonly prescribed for ocular conditions, may induce cataracts by increasing melanocyte activity in iris melanocytes, leading to iris hyperpigmentation (reported in approximately 7%–16% of patients). This pigmentation alteration may indirectly contribute to cataract formation by modifying the eye lens microenvironment, promoting oxidative stress, and facilitating free radical accumulation. Additionally, prostaglandin analogs may contribute to cataractogenesis through altered eye lens microenvironments or increased exposure to free radicals. Future research should integrate molecular investigations (such as apoptosis pathways in eye lens epithelial cells) with epidemiological studies to elucidate the underlying pathogenic mechanisms and identify high-risk populations.

Atorvastatin, a widely used 3-Hydroxy-3-methylglutaryl-Coenzyme A (HMG-CoA) reductase inhibitor, effectively reduces cholesterol synthesis and lowers low-density lipoprotein cholesterol (LDL-C) levels, thereby decreasing the risk of atherosclerosis and associated cardiovascular events. It is extensively prescribed for hypercholesterolemia, coronary heart disease, and both primary and secondary prevention of cardiovascular diseases (Liao and Laufs, 2005). Two pharmacovigilance studies have previously identified a significant association between statin use and an increased risk of cataracts (Erie et al., 2016; Klein et al., 2006). Consistent with these findings, our multivariate logistic regression analysis indicated that atorvastatin may be an independent risk factor for drug-induced cataracts (Odds Ratio (OR) [95% CI]: 12 [7.6–18]). However, existing research is not entirely consistent. Some studies have reported conflicting results, suggesting that statins may exert potential protective effects against cataracts, diabetic retinopathy, diabetic retinopathy progression, and non-infectious uveitis (Wagner and Rein, 2013; Kurinami et al., 2018). Given these contradictory findings, further research—ideally through large-scale randomized controlled trials (RCTs)—is necessary to clarify the relationship between statins and cataracts.

Zoledronic acid is a potent bisphosphonate primarily used to inhibit osteoclast activity, thereby reducing bone resorption. It is commonly prescribed for osteoporosis, bone metastases from cancer, and hypercalcemia (Major et al., 2001; Black et al., 2007). A study based on Optum and MarketScan databases reported a potential association between zoledronic acid treatment and an increased risk of cataracts (ROR [95% CI]: 12 [7.6–18]). The proposed mechanisms suggest that zoledronic acid may impair eye lens epithelial cell function by inducing local inflammation and protein denaturation, ultimately contributing to cataract formation (Lee et al., 2020). However, clinical evidence remains limited. Further research is warranted to elucidate the underlying mechanisms and explore the epidemiology of bisphosphonate-related cataracts.

Esomeprazole, a widely used proton pump inhibitor (PPI) for gastroesophageal reflux disease (GERD) and peptic ulcers, reduces gastric acid secretion by inhibiting H^+^/K^+^-ATPase activity (Qi et al., 2015). While its product labeling mentions occasional blurred vision as a potential adverse effect, comprehensive ophthalmic side effects remain poorly characterized. Our analysis suggests that esomeprazole may be an independent risk factor for cataracts. However, given the limited current evidence, further large-scale RCTs are warranted to elucidate this potential risk and assess its clinical implications comprehensively.

Atorvastatin, a widely used HMG-CoA reductase inhibitor, effectively inhibits cholesterol synthesis and lowers LDL-C levels, thereby reducing cardiovascular risk (Liao and Laufs, 2005). Two pharmacovigilance studies have previously reported a significant association between statin use and an increased risk of cataracts (Erie et al., 2016; Klein et al., 2006). Consistently, our multivariate logistic regression analysis identified atorvastatin as an independent risk factor for drug-induced cataracts (OR [95% CI]: 12 [7.6–18]). However, existing studies have yielded conflicting results regarding the relationship between statins and cataracts, with some evidence suggesting potential ocular protective effects against diabetic retinopathy, uveitis, and other ocular conditions (Wagner and Rein, 2013; Kurinami et al., 2018). Therefore, further RCTs are warranted to clarify this association.

Risankizumab, a monoclonal antibody targeting interleukin-23A (IL-23A), is primarily indicated for the treatment of plaque psoriasis, psoriatic arthritis, Crohn’s disease, and ulcerative colitis (Gordon et al., 2018). Previous pharmacovigilance studies have identified cataracts as a reported adverse reaction. Consistently, our multivariate logistic regression analysis suggested that Risankizumab may be an independent risk factor for cataracts. However, the precise pathogenic mechanisms remain unclear and warrant further investigation.

Interferon Beta-1A, indicated for multiple sclerosis, was also identified as potentially associated with drug-induced cataracts. Given the limited availability of direct clinical studies or case reports, further ocular monitoring in patients receiving Interferon Beta-1A is recommended to facilitate early detection and management of ocular complications.

Adalimumab, commonly used for the treatment of severe uveitis associated with juvenile idiopathic arthritis, has been reported to contribute to cataracts and glaucoma, leading to significant visual impairment (Lu et al., 2021; Fleischmann et al., 2022). However, in our study, the cataract signal associated with Adalimumab may represent a false positive, potentially influenced by underlying diseases or inadequate treatment responses. Therefore, further research is necessary to establish a definitive causal relationship between Adalimumab and cataracts.

Glucocorticoids have long been associated with cataract development, first reported by Wang (Wang et al., 2009). Since then, extensive evidence has demonstrated that both systemic and topical glucocorticoids, such as dexamethasone, prednisone, and hydrocortisone, significantly increase cataract risk (Black et al., 2016). Posterior subcapsular cataract (PSC) is the predominant clinical presentation of glucocorticoid-induced cataracts, distinguishing it from age-related and other cataract types. The underlying mechanism likely involves disruption of eye lens epithelial cell function, specifically interfering with proliferation, differentiation, migration, and apoptotic processes, ultimately leading to eye lens protein denaturation and opacity formation (Mishra et al., 2023).

Upadacitinib and Tofacitinib are selective Janus kinase (JAK) inhibitors that exert anti-inflammatory and immunomodulatory effects by inhibiting the JAK/signal transducer and activator of transcription (STAT) signaling pathway (Loftus et al., 2023). Recent pharmacovigilance studies have identified ocular adverse events, including cataracts, macular holes, and scleritis, as potential new safety signals not previously documented in Upadacitinib’s labeling (Wu et al., 2023). This finding aligns with our results, suggesting that the JAK/STAT pathway may contribute to cataract formation by influencing eye lens epithelial cell proliferation. Currently, no dedicated research has explored the relationship between Tofacitinib and cataracts; therefore, close ophthalmic monitoring in clinical practice is recommended.

Ibrutinib, a Bruton’s tyrosine kinase (BTK) inhibitor, irreversibly binds to BTK, thereby suppressing B-cell proliferation and survival (Li et al., 2021). In the RESONATE phase III multicenter trial, among approximately 400 patients with relapsed or refractory chronic lymphocytic leukemia (CLL) or small lymphocytic lymphoma (SLL) receiving Ibrutinib, 10% reported blurred vision, and 3% developed cataracts. Researchers have cautioned that the risk of ocular adverse events may increase with prolonged treatment (Li et al., 2021). Additionally, a case report suggested that Ibrutinib may penetrate the blood-aqueous barrier, altering the ocular microenvironment and causing platelet dysfunction and impaired fibrinolysis, potentially leading to anterior chamber fibrinoid syndrome following cataract surgery (Wang et al., 2025). Further prospective studies and molecular investigations are warranted to clarify the causal relationship between Ibrutinib and cataracts. Moreover, predictive risk models should be developed to optimize patient management.

Pomalidomide and Lenalidomide are primarily used for the treatment of relapsed or refractory multiple myeloma (Dimopoulos et al., 2021). Although no direct association between Pomalidomide and cataracts has been reported, a prospective study involving vitreoretinal lymphoma patients receiving intravitreal methotrexate (MTX), Rituximab, and Lenalidomide (*R* ^2^ regimen) as maintenance therapy identified notable ocular toxicities, including cataracts, suggesting a potential increase in ocular toxicity risk (Zhang et al., 2021). Our findings further support the hypothesis that both drugs may independently contribute to cataract risk. Future clinical research is warranted to elucidate their pathogenic mechanisms and assess cataract risk, aiming to optimize treatment strategies and ocular monitoring.

Sodium Oxybate, a central nervous system depressant and the sodium salt of γ-hydroxybutyric acid (GHB), is primarily used to treat narcolepsy-related symptoms, including cataplexy and disrupted nighttime sleep. Its mechanism of action involves modulating gamma-aminobutyric acid (GABA) receptor activity to enhance sleep quality and regulate the circadian rhythm (Avidan and Kushida, 2020). Currently, no studies have reported an association between Sodium Oxybate and ocular adverse events. However, our findings suggest a potential cataractogenic risk, highlighting the need for future ophthalmic safety monitoring and large-scale clinical studies to elucidate the associated risks and underlying mechanisms.

Tiotropium, a long-acting anticholinergic agent, is widely used for the long-term management of chronic obstructive pulmonary disease (COPD) and asthma (Blair, 2019). Currently, limited evidence directly links Tiotropium to cataractogenesis. Cataract formation is typically influenced by multiple factors, including aging, UV exposure, diabetes, and prolonged corticosteroid use (Yong et al., 2022). Tiotropium exerts its effects by antagonizing M3 cholinergic receptors, thereby reducing airway smooth muscle constriction. However, this mechanism does not directly impact eye lens metabolism or structure. Although no conclusive evidence currently supports a causal relationship, regular ophthalmologic examinations during prolonged Tiotropium treatment are advisable. Future large-scale longitudinal studies are needed to further investigate the potential cataractogenic risk and underlying mechanisms of Tiotropium, aiming to optimize drug safety evaluations and clinical management strategies.

This study identified a median onset time of drug-induced cataracts as 449 days (interquartile range: 150–901 days), with over 75% of cases occurring within 747 days following drug initiation. This finding suggests a significant latency period for drug-induced cataracts, accompanied by considerable inter-individual variability.

Previous studies support this observation. For instance, Cumming et al. demonstrated that prolonged inhaled glucocorticoid use is associated with an increased cataract risk, typically requiring months to years of cumulative exposure (Wang et al., 2009). Similarly, studies by Klein et al. and Smeeth et al. highlighted prolonged intervals between drug exposure and cataract onset, ranging from months to years (Smeeth et al., 2003). Collectively, these findings align with our results, indicating a slow progression and high variability in the onset timing of drug-related cataracts. Consequently, patients undergoing long-term treatment with high-risk medications require continuous ophthalmologic monitoring to facilitate early detection and intervention, ultimately reducing the risk of vision impairment associated with drug-induced cataracts.

Nevertheless, this study has several limitations. First, the FAERS relies on voluntary submissions from healthcare professionals, patients, and pharmaceutical companies, which may lead to underreporting or reporting bias. Second, as FAERS was not specifically designed to establish causality, its case-report-based data lack standardized follow-up and control groups, limiting the ability to draw direct causal inferences between drug exposure and cataract development (Li et al., 2025a). This limitation is a common challenge in pharmacovigilance and observational cohort studies. Additionally, FAERS often contains missing information, including specific drug dosages, patient comorbidities, cataract onset timing, and other critical clinical variables, which may compromise analytical accuracy (Li et al., 2025b). Furthermore, FAERS does not specify cataract subtypes (e.g., cortical, nuclear, or posterior subcapsular cataracts), reducing the specificity of study outcomes. Therefore, caution is warranted when interpreting data-mining results, and conclusions should be corroborated through comprehensive evidence-based assessments to ensure clinical reliability and applicability.

## Conclusion

This study utilized FAERS data to compile a comprehensive list of drugs potentially associated with drug-induced cataracts. The findings provide valuable insights for the early identification of drug-related cataracts and serve as a reference for future research on the pathogenesis of drug-induced cataracts. However, all reported findings require further validation through additional clinical studies and animal experiments.

## Data Availability

The original contributions presented in the study are included in the article/Supplementary Material, further inquiries can be directed to the corresponding authors.

## Glossary

FDA: U.S.Food and Drug Administration
FAERS: FDA Adverse Event Reporting System
ROR: Ratio of Odds Ratios
LASSO: least absolute shrinkage and selection operator
IQR: Interquartile Range
WHO: World Health Organization
READUS-PV: Recommendations for the Evaluation of Adverse Drug Reaction Signals Using Individual Case Safety Reports
MedDRA: Medical Dictionary for Regulatory Activities
PRIMARYID: Primary Identifier
ISRs: Individual Case Safety Reports
CI: Confidence Interval
PostgreSQL: Post-Ingres Structured Query Language
ROC-AUC: Receiver Operating Characteristic - Area Under the Curve
HMG-CoA: 3-Hydroxy-3-methylglutaryl-Coenzyme A
LDL-C: Low-Density Lipoprotein Cholesterol
OR: Odds Ratio
RCTs: Randomized Controlled Trials
PPI: Proton Pump Inhibitor
GERD: Gastroesophageal Reflux Disease
IL-23A: Interleukin-23A
PSC: Posterior Subcapsular Cataract
JAK: Janus kinase
STAT: Signal Transducer and Activator of Transcription
BTK: Bruton’s Tyrosine Kinase
CLL: Chronic Lymphocytic Leukemia
SLL: Small Lymphocytic Lymphoma
MTX: Methotrexate
GHB: γ-hydroxybutyric acid
GABA: Gamma-Aminobutyric Acid
COPD: Chronic Obstructive Pulmonary Disease.
